# Senescence-associated ultrastructural features of long-term cultures of induced pluripotent stem cells (iPSCs)

**DOI:** 10.18632/aging.101309

**Published:** 2017-10-23

**Authors:** Fiorella Colasuonno, Rossella Borghi, Alessia Niceforo, Maurizio Muzzi, Enrico Bertini, Andrea Di Giulio, Sandra Moreno, Claudia Compagnucci

**Affiliations:** ^1^ Department of Science, LIME, University “Roma Tre”, Rome 00146, Italy; ^2^ Department of Neuroscience, Unit of Neuromuscular and Neurodegenerative Diseases, Laboratory of Molecular Medicine, Bambino Gesu' Children's Research Hospital, IRCCS, Rome 00146, Italy

**Keywords:** iPSCs, FIB/SEM, aging, mitochondria, cell-cell contacts, autophagy

## Abstract

Induced pluripotent stem cells (iPSCs) hold great promise for developing personalized regenerative medicine, however characterization of their biological features is still incomplete. Moreover, changes occurring in long-term cultured iPSCs have been reported, suggesting these as a model of cellular aging. For this reason, we addressed the ultrastructural characterization of iPSCs, with a focus on possible time-dependent changes, involving specific cell compartments. To this aim, we comparatively analysed cultures at different timepoints, by an innovative electron microscopic technology (FIB/SEM). We observed progressive loss of cell-to-cell contacts, associated with increased occurrence of exosomes. Mitochondria gradually increased, while acquiring an elongated shape, with well-developed *cristae*. Such mitochondrial maturation was accompanied by their turnover, as assessed by the presence of autophagomes (undetectable in young iPSCs), some containing recognizable mitochondria. This finding was especially frequent in middle-aged iPSCs, while being occasional in aged cells, suggesting early autophagic activation followed by a decreased efficiency of the process with culturing time. Accordingly, confocal microscopy showed age-dependent alterations to the expression and distribution of autophagic markers. Interestingly, responsivity to rapamycin, highest in young iPSCs, was almost lost in aged cells. Overall, our results strongly support long-term cultured iPSCs as a model for studying relevant aspects of cellular senescence, involving intercellular communication, energy metabolism, and autophagy.

## INTRODUCTION

Starting from the development of induced pluripotent stem cells in 2006 [1], great advances have been accomplished in the field of stem cell biology and in regenerative medicine. Thanks to this technology, it is possible to gain information on the molecular me-chanisms underlying human pathologies, especially those hardly reproducible in a cellular or animal model, as some neurological disorders [2]. Moreover, iPSCs are used for drug screening and for the development of cellular therapies. However, despite the need to fully characterize iPSCs, prior to their safe use in trans-lational medicine, ultrastructural studies on iPSCs are still few [3-5]. Further, information on possible morphological changes occurring as a function of culturing time are at present unexplored. For this reason, we performed ultrastructural studies focusing on the nuclear and cytoplasmic features during *in vitro* maintenance in aerobic environment. The current concept that iPSCs can be indefinitely maintained in culture has been recently challenged by studies on the epigenetic status, the nucleoskeleton, and the mito-chondrial functionality in long-term cultured iPSCs (up to one year) in incubators with O_2_ 21%; CO_2_ 5%, which however do not mimic the physiological stem cell niche [6-8]. Moreover, tumorigenic potential is demonstrably increased in “aged” iPSCs [6]. We analyzed ultra-structural changes related to cell senescence, comparing iPSCs kept in culture for one month (young-iPSCs, y-iPSCs), six months (middle-aged-iPSCs, ma-iPSCs) and one year (aged-iPSCs, a-iPSCs), by using Focussed Ion Beam/Scanning Electron Microscopy (FIB/SEM). Even though FIB/SEM technology has been developed two decades ago and successfully applied to materials sciences, biological applications started more recently [9, 10]. Among the few cell biology studies which so far took advantage of this innovative approach, no one is available on stem cells.

Based on electron microscopic data, showing, among others, mitochondrial abnormalities, and suggesting their altered turnover by autophagic process, we also investigated the distribution of vesicular trafficking markers and autophagic players, by immunofluo-rescence and immunoblotting. The ultimate aim of this work is to improve knowledge in the stem cell biology field, bringing new evidence to support long-term cultured iPSCs as a valuable model for dissecting the mechanisms of cell senescence.

## RESULTS

### Ultrastructural characterization of y-, ma- and a-iPSCs

**Figure 1:**
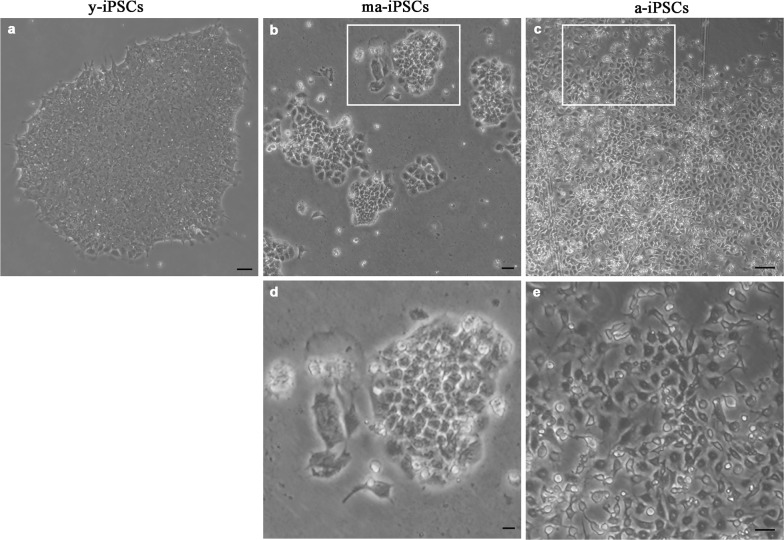
Bright field images, acquired at the inverted microscope of y-iPSCs (**a**), ma-iPSCs (**b**,**d**) and a-iPSCs (**c**,**e**). Scale bars, 20 μm (**a**-**c**), 10 μm (**d**,**e**).

The morphological aspect of iPSCs, as observed in the inverted microscope, shows obvious differences among cultures at different timepoints. While y-iPSCs appear as well-organized colonies with regular margins, aged iPSCs display progressive loss of their ability to form colonies, and cells are found in small groups with irregular shapes (ma-iPSCs) or isolated (a-iPSCs) (Figure 1). Noteworthy, aging iPSCs maintain their pluripotency features, as already demonstrated [8]. Based on these observations, we explored ultrastructural features of iPSCs using a Dualbeam FIB/SEM Helios Nanolab microscope (FEI, Hillsboro, USA), an instrument that combines an electron beam (SEM column) with a focused gallium ion beam (FIB column), oriented at 52°, and focusing on the same area of the specimen. This innovative approach allows obtaining ultrastructural information without ultra-microtomy and TEM analysis. FIB column is used as a nanoscalpel to perform cross sections in a specific region of the sample, while SEM column is used to obtain high resolution TEM-like images of the cells thanks to the backscattered electrons detected at the level of the milled surface.

**Figure 2:**
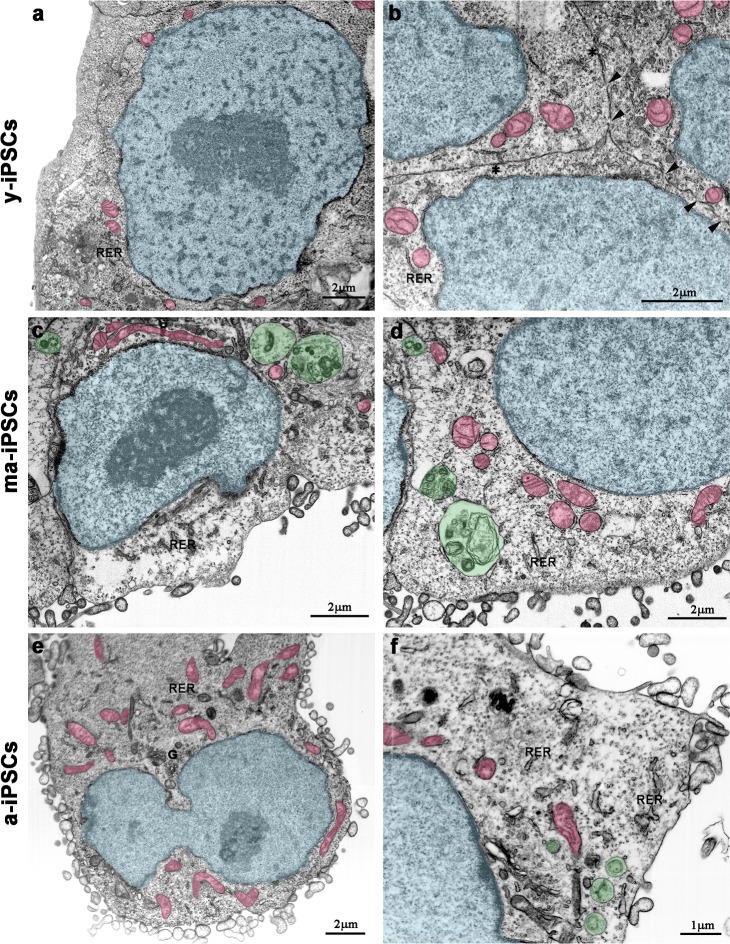
FIB/SEM micrographs of y-iPSCs (**a**,**b**), ma-iPSCs (**c**,**d**) and a-iPSCs (**e**,**f**). Images have been electronically colored to highlight nuclei (in blue), mitochondria (in pink), and autophagosomes (in green). Arrowheads, gap junctions; asterisks, tight junctions; G, Golgi apparatus; RER, rough endoplasmic reticulum.

**Figure 3:**
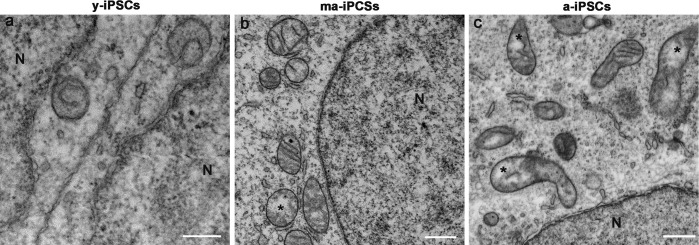
FIB/SEM micrographs of y-, ma- and a-iPSCs showing changes in mitochondrial structure during culturing time. Mitochondria with disorganized *cristae* are indicated by the asterisks in ma- and a-iPSCs. N, nuclei Scale bars, 1μm.

Electron microscopy confirms light microscopic data, providing further details on culturing time-dependent morphological features. In y-iPSCs, FIB/SEM images show extended cell-to-cell contacts, including tight and gap junctions (Figure 2b). In their cytoplasm, a small number of mitochondria with round shaped morphology and poorly developed *cristae*, mainly localized around the euchromatic nucleus are detected (Figure 2a, b and Figure 3a).

**Figure 4:**
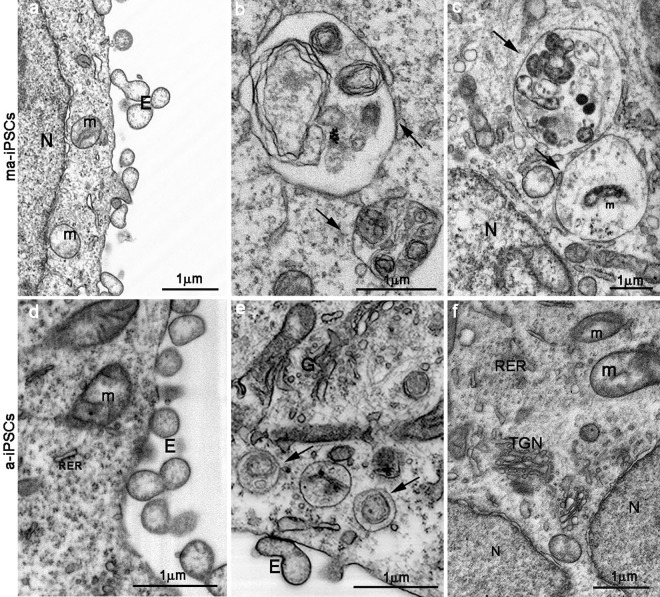
FIB/SEM images showing ultrastructural details of ma- (**a**-**c**) and a-iPSCs (**d**-**f**). Arrows in b,c,e indicate autophagosomes containing mitochondria (m) or other partially digested cytoplasmic material. E, exosomes; N, nuclei; (TGN, trans-Golgi network; RER, rough endoplasmatic reticulum).

In ma- and a-iPSCs, electron microscopy shows irregularly shaped plasma membranes, close to which spheroidal vesicles are present (Figure 2c-f). These vesicles, likely corresponding to exosomes, based on their appearance and size (40-200 nm in diameter), are frequently observed in ma-iPSCs and even more numerous in a-iPSCs (Figure 2c-f; Figure 4a, d) [11]. The nucleus of the ma- and a-iPSCs is often poly-morphic, with irregular margins, blebs or invaginations (Figure 2e, f), while mitochondria, dispersed in the cytoplasm, display an elongated shape, with well-developed *cristae* (Figure 2c-f; Figure 3b, c). Noteworthy, mitochondrial alterations, including the presence of disorganized cristae and abnormal inner spaces membrane, are occasionally encountered in ma-iPSCs, while representing a frequent finding in a-iPSCs (Figure 2c-f; Figure 3b, c). Additional differences from y-iPSCs con-cern vesicular trafficking features in ma- and a- iPSCs.

These consist of an abundant Golgi apparatus -especially trans-Golgi network-, associated with numerous endo-lysosomic vesicles and double-membrane limited autophagosomes. The latter, espe-cially frequent in ma-iPSCs, often contain recognizable mitochondria or other partially digested cytoplasmic components (Figure 4).

### Autophagy regulation in y-, ma-, and a-iPSCs

Ultrastructural data, demonstrating the presence of endo-lysosomal and autophagic vesicles in ma- and a-iPSCs, prompted us to further investigate the activation of autophagy through the analysis of specific markers.

**Figure 5:**
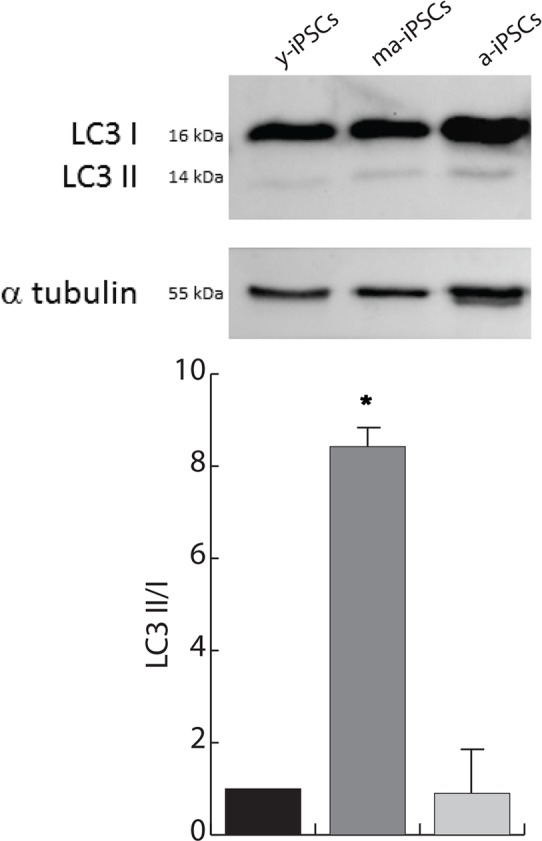
Autophagy is increased in ma-iPSCs when compared with y- and a-iPSCs. Western blot analysis of LC3 I/II, and alpha-tubulin as loading control is shown. Bar graph shows the LC3–II/I ratio and represents the mean± SD of three experiments.

To understand whether the autophagic flux is modified during iPSCs aging, we performed western blot analysis for microtubule-associated protein 1A/1B-light chain (LC3). Its conversion from the soluble form (LC3 I) to the phosphatidylethanolamine-conjugated form (LC3 II), which is recruited to autophagosomal membranes, indicates active autophagic process. Consistent with FIB/SEM data, remarkable accumulation of LC3 II is found in ma-iPSCs (Figure 5), thus accounting for increased autophagy, while in a-iPSCs the LC3 II/I ratio decreases, reaching levels only mildly higher than y-iPSCs.

**Figure 6:**
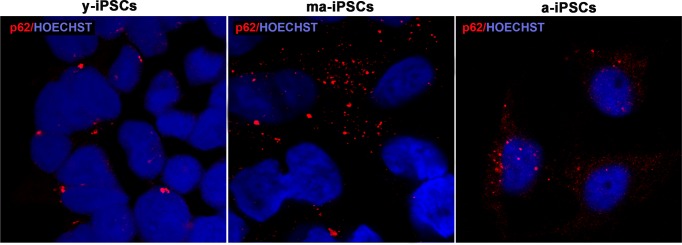
Confocal microscopic images of y-, ma- and a-iPSCs marked with p62 (in red) and Hoechst (in blue) showing abundant granular p62 immunopositivity in ma-iPSCs and in a-iPSCs when compared with y-iPSCs.

For a morphological characterization of autophagic process in iPSCs, we performed immunofluorescence experiments using p62, Beclin1 and Lamp2 markers. The former protein (also known as Sequestosome-1), encoded by *SQSTM1* gene, serves as a scaffold for ubiquitinated proteins to be degraded thanks to its binding to LC3, and accumulates at depolarized mito-chondria, leading to mitophagy [12, 26]. Confocal images show an increased trend of p62 in ma-iPSCs and in a-iPSCs, when compared with y-iPSCs, suggesting initial mitochondrial damage (Figure 6 and Suppl. Figure 1a).

**Figure 7:**
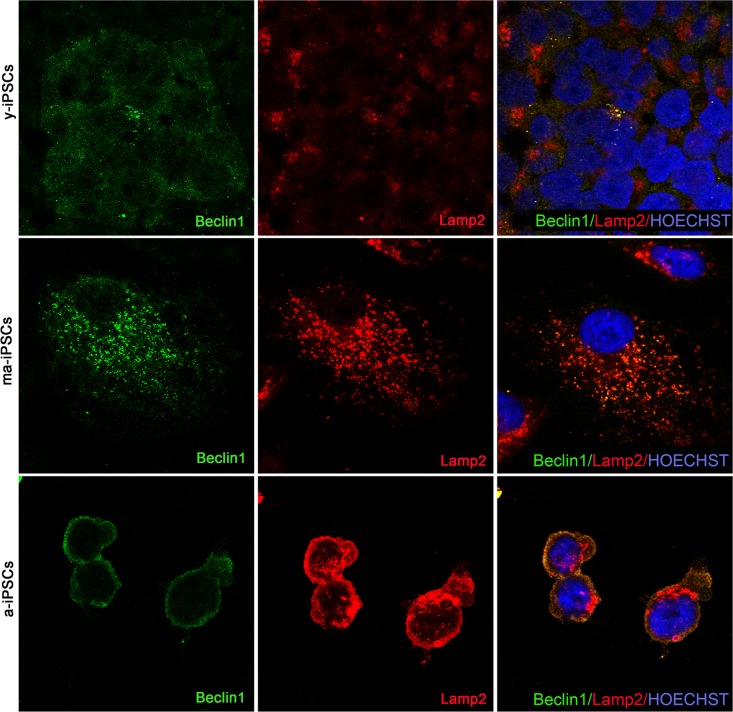
Confocal microscopy of y-, ma- and a-iPSCs immunostained with Beclin1 (in green), Lamp2 (in red) and Hoechst (in blue). In ma-iPSCs the central cell shows a granular distribution of Lamp2, while the arrow indicates a cell with a perinuclear staining similar to those observed in a-iPSCs.

Beclin1, encoded by *BECN1* gene is essential for the initial phases of the autophagic process, as it interacts with several cofactors leading to the formation of Beclin1-Vps34-Vps15 complex, necessary for phagosome assembly. Immunofluorescence analysis shows dot-like distribution of the protein and significantly higher levels in a-iPSCs when compared with either y- or ma-iPSCs (Figure 7 and Suppl. Figure 1b).

**Figure 8:**
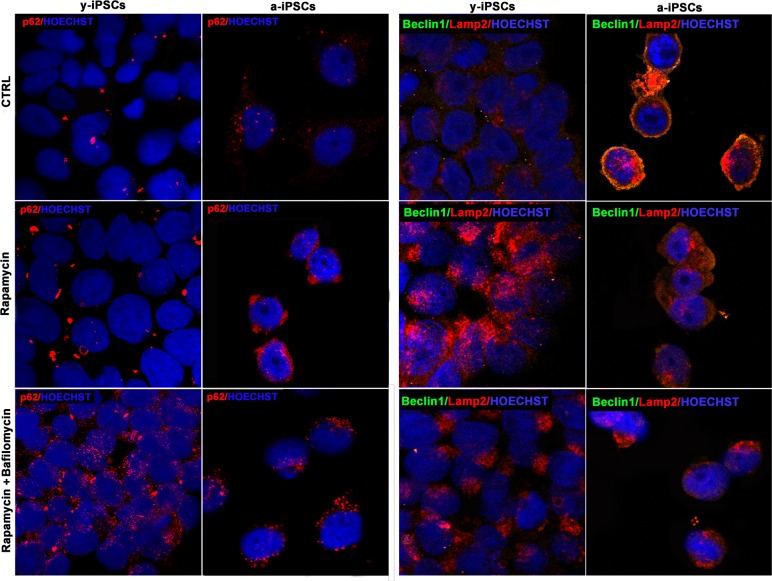
Confocal microscopy of y- and a-iPSCs stained with anti-p62 antibody (in red), anti-Beclin1 (in green), Lamp2 (in red) and Hoechst (in blue), treated with rapamycin alone or in combination with bafilomycin.

Immunofluorescence analysis of Lamp2, a membrane glycoprotein associated to the lysosomes, playing a fundamental role in the fusion of these organelles with autophagosomes, shows the typical granular distribu-tion, compatible with its localization on the above vesicles. Intriguingly, in a-iPSCs, Lamp2 is also localized at the plasma membrane, forming clusters in the cytoplasm, reminiscent of endo-lysosomal derived exosomes (Figure 8). Quantitating immunofluorescence data demonstrate significantly elevated levels of the protein in ma- and a-iPSCs compared with y-iPSCs (Suppl. Figure 1c).

We then addressed the possible induction of autophagy in a-iPSCs, as compared to y-iPSCs, by rapamycin treatment, with or without bafilomycin, the latter used as an inhibitor of autophagosome-lysosome fusion. Interestingly, while y-iPSCs display high rapamycin-induced autophagic activity, a-iPSCs are relatively little responsive to the drug, as assessed by p62, Beclin1 or Lamp2 immunofluorescence. Nevertheless, co-treat-ment with bafilomycin, which stabilizes autophago-somes, thus preventing their degradation, results in some increase of the examined markers even in a-iPSCs (Figure 8). It should however be noted that rapamycin treatment, independent of the presence of bafilomycin, results in a change of Lamp2 distribution in a-iPSCs, with a shift from a periplasmic to a deeper cytoplasmic compartment, possibly related to modifications to vesicular trafficking system.

## DISCUSSION

In the last decade, the knowledge of pluripotent stem cell biology has considerably increased [13, 14]. However, while genetic and epigenetic aspects are being widely explored [15], ultrastructural studies dealing with iPSCs are still few and focused on specific cellular features [3-5, 16], despite the fact that morphology represents a discriminating parameter of any cellular model. In fact, morphology of iPSC colonies is the primary aspect taken into consideration, in order to proceed with the colony picking from the heterogenous fibroblast culture. For this reason, we decided to analyze iPSCs ultrastructure by Dual beam FIB/SEM, an innovative technology previously utilized by our group for different purposes [17, 18]. We particularly focused on culturing time-dependent changes in iPSCs features, based on previous studies suggesting that iPSCs cannot be maintained in culture indefinitely, as these cells undergo demonstrable age-related molecular alterations [7, 8]. Our present results, while supporting the above concept, importantly add novel information on iPSCs inter- and intra-cellular features, including colony-forming ability, mito-chondrial status and autophagic pathway. Moreover, by including in the study three timepoints, i.e., y-, ma-, and a-iPSCs, we could characterize age-related changes in a developmental perspective.

Overall, our data confirm that iPSCs progressively lose their capacity to grow as colonies, as assessed by light and electron microscopy, showing limited ability of ma- and a-iPSCs to develop extended cell-cell contacts. In fact, gap-junctions are hardly recognized in ma- and a-iPSCs specimens, differently from y-iPSCs, where they are readily identified, in agreement with literature data [5]. This strongly suggests a decreased ability of cells to communicate to each other. To this respect, it is worth noting that ma- and a-iPSCs display spheroidal vesicles close to plasma membranes, possibly corresponding to exosomes, based on their shape and size. The presence of exosomes is not reported in embryonic stem cells (ESCs) or iPSCs, while recent studies demonstrated that mesenchymal stem cells (MSCs) can produce exosomes as vectors of paracrine signals, with a protective role [19-21]. Thus, we hypothesize that these vesicles are released as an alternative mechanism of intercellular communication, consequent to age-related decreased capacity to form cell-to-cell contacts. Further studies are needed to verify this hypothesis, as well as to explore whether exosomal vesicles may serve as a mean of cytoprotection, even in iPSCs. The high number of presumed exosomes observed in a-iPSCs is consistent with their abundant Golgi apparatus, particularly *trans*-Golgi network. Indeed, this cytoplasmic compartment, scarce in y-iPSCs, can be linked to exosomal vesicles deriving from the endocytic compartment of the cell [11]. On the other hand, endo-lysosomal involvement in culturing time-dependent changes of iPSCs is also witnessed by altered macro-autophagic activity. This was assessed by WB and confocal microscopy following immunofluorescence with autophagy-related markers, namely LC3, p62, Beclin1 and Lamp2. Interestingly, ma- and a-iPSCs showed progressively higher levels of the latter three markers, when compared to y-iPSCs, strongly arguing for autophagic activation. Such process, well visualized in FIB/SEM images, seems to lead specifically to mitochondria turnover, since these organelles are sometimes recognized inside the double-membrane limited autophagosomes. It should be noted, however, that autophagosome occurrence is more frequent in ma- than in a-iPSCs, consistent with LC3II protein levels, showing remarkably higher levels in ma-iPSCs than in a-iPSCs. Thus, while being strongly induced, successful completion of the autophagic process may be impaired. In line with this concept, the localization of Lamp2 at the plasma membrane in a-iPSCs argues for different roles of this endo-lysosomal protein, possibly linked with exosome formation and release [33, 34]. By contrast, in ma-iPSCs Lamp2 immunoreactive granules are strongly reminiscent of autophagolysosomes. Closely related to these findings are the ultrastructural changes detected in mitochondrial population. Differently from y-iPSCs, where the organelles are scarce and show a perinuclear distribution as reported by [16, 22], in ma- and even more in a-iPSC mito-chondria are numerous and dispersed in the cytoplasm. Moreover, progressive mitochondrial maturation, involving size increase, shape elongation and *cristae* development, is observed during culturing time. These results are in line with previous data from ours and other Authors' groups reporting. increased mitochondrial biogenesis [7, 23], and are possibly due to adaptation of the cells to the culture environment (5% CO_2_ and 21% O_2_). In fact, stem cells have a glycolytic metabolism [16, 24, 25] that is not suited to the aerobic environment and for this reason we consider compulsory to deepen the study on iPSCs aging using low-oxygen tension cell culture environment (3% CO_2_ and 5% O_2_). In fact, as we recently reported [7, 8], the aerobic environment could be responsible for the conversion from glycolytic to oxidative metabolism, as an adaptation to the high O_2_ level. To avoid this change, potentially leading to aging, it is important to maintain iPSCs in an anaerobic environment and this condition may be a path to slow down the pace of iPSCs aging in culture. Specifically, in a-iPSCs, we frequently detected abnormal mitochondrial features, including fragmented cristae and dilated inner space. This finding may well be related to the above suggested impairment of mitophagy, particularly in its final steps. Indeed, the granular cytoplasmic pattern shown by p62 supports this hypothesis, since this protein has been observed in depolarized mitochondria, probably for the recruiting of the damaged organelles toward the autophagosomes [26]. Besides, treatment with mTOR inhibitor rapamycin seems inefficacious in a-iPSCs, contrary to y-iPSCs, which are highly responsive to the drug, revealing a disrupted mTOR signaling pathway, in accordance with mechanisms of cellular senescence already described [27]. Importantly, ultrastructural analysis of aging iPSCs also reveals progressive alterations of the nuclear compartment, which appears polymorphic, with irregular margins, blebs and invaginations, if compared to the regularly shaped nuclei of y-iPSC, as previously described [28]. Our observations well correlate with the latest study by Petrini et al. [8], describing nucleoskeletal dysmor-phisms and altered expression of Lamin A/C in a-iPSCs. Moreover, the relevance of mitochondrial autophagy, or mitophagy, in the context of stem cell biology has been demonstrated by a recent study [29], where mitophagy-deficient iPSCs colonies were obtained with low efficiency and they had a significant tendency to spontaneously differentiate and form heterogeneous cell populations. Importantly, during conversion of somatic cells to iPSCs, mitophagy is responsible for mitochondrial rejuvenation. It is therefore possible that mitophagy also plays a crucial role in iPSCs aging and that the decreased autophagic response in a-iPSCs could be one reason why mito-chondria appear more mature and more numerous. Another reason for the augmented number of mitochondria may be increased biogenesis of these organelles. During reprogramming, genes involved in mitochondrial biogenesis (TFAM, NRF1) are gradually upregulated [30] and during aging iPSCs show an increase of the same genes [7]. This suggests a dynamic change in mitochondria biogenesis in both processes of rejuvenation of somatic cells in iPSCs and aging of these latter. The study by the lab of Dr. Wong [30] investigated the consequences of mitochondrial defective functionality in stem cell biology, and used iPSCs from patients with Leber's hereditary optic neuropathy (LHON) as a model to study the effects of oxidative phosphorylation (OXPHOS) defects in reprogramming. This study demonstrated that iPSCs can be obtained from LHON- fibroblasts with normal efficiency, thus suggesting that iPSC reprogramming can tolerate a certain degree of OXPHOS defects. A successful iPSC model for translational medicine was obtained by Wong et al. [31], who used cybrid technology to replace mutated mitochondrial DNA (mtDNA) from LHON-specific fibroblasts with wildtype mtDNA. The authors obtained mutation-free iPSCs and in these cybrid corrected iPSC-derived retinal ganglion cell, the cell death phenotype was res cued. The usefulness of the iPSC model is also offered by the work of Crombie et al. [32], in which, Friedreich's ataxia (FRDA) iPSCs have been differentiated into cardiomyocytes to be used for future high throughput compound screening. In line with these iPSCs models, we suggest that our aging model provides an opportunity to investigate stem cell properties and the aging biology of stem cells. Studies investigating the molecular determinants of aging in stem cells may account on a well characterized model which could be used for high throughput compound screening and it could contribute to the identification of new treatments for human aging.

## CONCLUSIONS AND PERSPECTIVES

**Figure 9:**
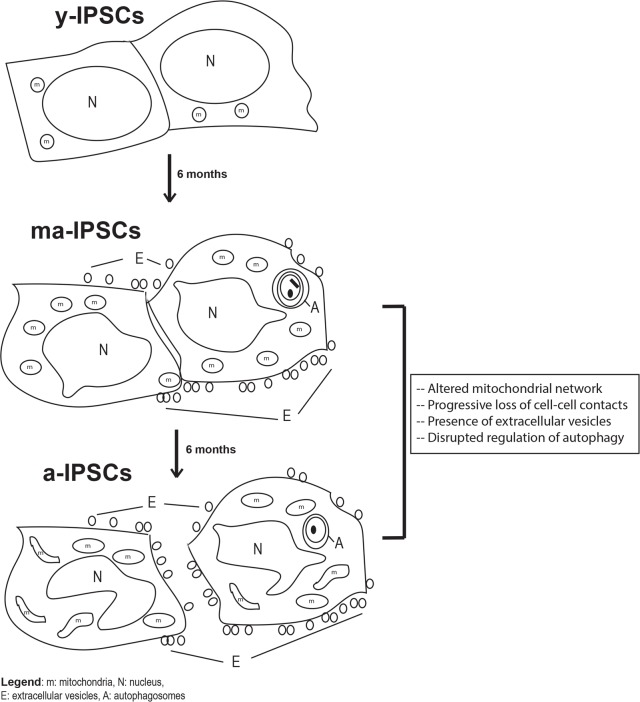
Schematic diagram of biological features observed in y-iPSCs, ma- (following 6 months of culture) and a-iPSCs (In particular, we highlighted the mitochondrial disruption, the progressive loss of cell- cell contacts, the presence of autophagosomes and extracellular vesicles with aging. m, mitochondria; N, nucleus; E, extracellular vesicles; A, autophagosomes.

In conclusion, our study brings new knowledge on the aging process occurring in long-term iPSCs cultures, an issue that has so far been little explored, despite its crucial understanding for future clinical applications. In fact, the present manuscript represents a further development of our previous works [7, 8], where the dogma claiming that iPSCs can be indefinitely maintained in culture was first challenged. Our novel morphological, ultrastructural and molecular data on ma- and a-iPSCs consistently suggest a progressive cellular senescence phenotype in iPSCs, including i) alteration of cell-cell contacts, with exosomes production, as an alternative instrument of intercellular communication; ii) mitochondrial maturation, followed by their dysfunction; iii) autophagic activation, followed by loss of efficiency (Figure 9). The hypothesized altered functionality of such key cell processes opens the way to further work aimed at characterizing energy metabolism pathways in aged iPSCs. The ultimate aim of such studies would be to identify novel experimental strategies for maintenance of long-term iPSCs cultures, avoiding senescence processes which clearly hinder the use of these cells for clinical purposes. To this respect, it is noteworthy mentioning that preliminary results from our lab suggest that when kept under hypoxic conditions, iPSCs apparently ameliorate their senescent phenotype.

Besides, our model appears suitable to studies on pathological conditions, particularly tumors, as it shares important similarities with cancer cells, namely increased proliferation and loss of the ability to form cell-cell contacts. To this respect, it is worthwhile mentioning that augmented Lamp2 levels at the plasma membrane have been reported in tumor cells, as con-sequence of altered inter-cellular communication, leading to loss of contact inhibition [33-35], in a similar fashion that we observe in a-iPSCs. Future development of the present work may therefore include further characterization of structure and regulation of cell junctions and adhesion mechanisms in iPSCs, by molecular/morphological combined approaches, also taking advantage of the great potential of FIB/SEM in providing serial tomographic images, which can be reconstructed to create 3D pictures and in allowing correlative microscopy [36, 37].

## MATERIALS AND METHODS

### Cell Culture

#### Derivation of human iPSCs (h-iPSCs)

Human fibroblasts obtained from skin biopsies of two healthy adult male individuals were purchased from Coriell Institute (USA, Cod GM23338, derived from a 55 years old male, and GM25256, derived from a 30 years old male). The iPSCs were derived from human fibroblasts and reprogrammed using the non-integrating episomal technology (Minicircle DNA and mc-iPS Cells, Euroclone, Cat. # SC301A-1).

#### Maintenance of iPSCs

Following thawing, iPSCs were grown on MEFs (Life Technologies) for two passages and then in feeder-free condition using Matrigel (BD Biosciences) in mTeSR1 (Stemcell Technologies). When iPSCs were 70–80% confluent, they were passaged 1:4 and transferred to new wells in feeder-free condition and incubated at 37°C, 5% CO2, the medium was changed every day and the cells split every three days. For the present study, we considered as y-iPSCs, ma-iPSCs or a-iPSCs those cultures maintained for 1, 6, or 12 months, respectively. General morphological characterization of cell cultures was assessed by their observation at the inverted microscope Olympus IX70, equipped with IAS 2000 image capturing software.

### Preparation of samples for ultrastructural analysis

Human iPSCs were cultured on Matrigel (BD Biosciences) in mTeSR1 (Stemcell Technologies). They were transferred to Chamber Slide™ (Lab-Tek®) and fixed in 0.5% glutaraldehyde and 2% paraformaldehyde in 0.1M cacodylate buffer for 50 minutes, then post fixed in 1% osmium tetroxide in 0.1M cacodylate buffer for 45 minutes, in the dark. Samples were washed for 30 minutes and contrasted with 1% uranyl acetate, then gradually dehydrated in ethanol. All the steps of the above procedure were performed at 4 °C. Human iPSCs were infiltrated with a mixture of 100% ethanol and epoxy embedding medium (Sigma-Aldrich™) and then embedded in the same resin allowing specimens to polymerize at 60 °C, for 3 days.

### Focussed Ion Beam/Scanning Electron Microscopy (FIB/SEM)

Samples were analysed by the Dualbeam FIB/SEM Helios Nanolab (FEI, Hillsboro, USA). Dualbeam instrument combines one electron beam (SEM column) and one ion beam (FIB column), oriented at 52°, and focused on the same point of the sample, thus enabling the operator to selectively ablate in a nanometer scale a previously marked region of the sample by using a focused ion current from a gallium source. The milling process can be interrupted every few nanometers to take high-resolution images of cross sections by the SEM column. Resin-embedded iPSCs were mounted on stubs by using a self-adhesive carbon disk and gold sputtered by an Emithech K550. Regions of interest were cross-sectioned by the focused gallium ion beam operated at 30 kV and 6.5 nA. Pictures of each cross-section were acquired at a working distance of 2 mm using backscattered electrons (BSE) and a through-the-lens (TLD) detector in immersion mode with an operating voltage of 2 kV and an applied current of 0.17 Na.

### Cell treatments

Rapamycin (Rapamycin #R8781, Sigma Aldrich) has been used to treat iPSCs at a final concentration of 500 nM at 37°C for 5 hours. Following Rapamycin treatments, bafilomycin (Bafilomycin #B1793, Sigma Aldrich) has been administered to the iPSCs at a final concentration of 100 nM at 37°C for 3 hours. Following the treatments, the cells have been fixed in 100% methanol for 5 minutes at −20°C and the cells washed twice in PBS 1X for 10 minutes each.

### Immunofluorescence analyses

For the immunofluorescence studies, the fixed cells have been treated with the blocking and permeabilizing solution (5% BSA, 0.1% Triton X-100 in PBS) for 1 hour at room temperature. Following this, the cells have been incubated with the primary antibody of interest, Beclin1 (SC-10086, Santa Cruz Biotechnology) 1:100 at 4°C overnight, LC3 (#2775, Cell Signalling Technology) 1:100 at 4°C overnight, Lamp2 (SC-18822, Santa Cruz Biotechnology) 1:100 at 4°C overnight, SQSTM1/p62 (#ab56416, Abcam) 1:75 for 1 hour at room temperature. The secondary antibodies used are Alexa Fluor 488 or Alexa Fluor 555 (Invitrogen) diluted 1:500 in PBS 1X for 1 hour. Then the nuclei have been stained with Hoechst 1ug/ml (#33342, Invitrogen) 1:10000 in PBS 1X for 10 minutes at room temperature. The coverslips have been mounted using 1:1 PBS/Glycerol.

### Confocal microscopy

Confocal optical sectioning is performed with on a Leica TCS-SP8X laser-scanning confocal microscope (Leica Microsystems, Mannheim, Germany) equipped with a white light laser (WLL) source and a 405nm diode laser. Sequential confocal images are acquired using a HC PLAPO 63x oil immersion objective (1.40 numerical aperture, Leica Microsystems). The images were assembled in Adobe Photoshop CS6 software (Adobe Systems Inc., San Jose, CA).

### Western blot analysis

Cells were mixed at 4 °C with lysis buffer containing RIPA 1X (RIPA Buffer #9806, Cell Signaling), protease inhibitor 1X (PierceTM Protease and Phosphatase Inhibitor Mini Tablets #88668, Thermo scientific) and 0,5 μM of sodium orthovanadate (#S6508, Sigma). After 10 minutes the mix was centrifuged at  4°C for 10 min at 13500 G and the supernatant, containing the protein extracts, was collected. An equal amount of protein extracts were applied onto 12% SDS-acrylamide gel electrophoresis and proteins transferred onto a nitrocellulose membrane. The membrane was blocked with 5% blocking agent (Amersham ECL Blocking Agent, #RPN2125) in TBST for 1 h, at room temperature, and probed overnight with monoclonal anti-LC3 antibody (#12741, Cell Signaling) (1/1000) and anti-α tubulin antibody (#PA5-19489, Thermofisher Scientific) (1/1000). Primary antibody incubation was followed by goat anti-rabbit (#111 035 003, Jackson Immuno-research Laboratories INC) secondary antibodies (1:10000) for both the primary ones. Immunoreactive proteins were visualized and quantified by chemiluminescence (Molecular Imager ChemiDOC XRS+, Biorad; Software ImageLab).

### Statistical analysis

Results are referred from at least three independent experiments from the two iPSC lines obtained from healthy individuals (in 5% CO2, 21% O2). Data are expressed as mean and standard deviation. Comparisons between groups are performed by two-tailed unpaired student's t-test and p values <0.05 were considered statistically significant (*). (**) indicates p values inferior to 0.005. Data are analyzed using Windows XP Excel.

## SUPPLEMENTARY MATERIAL FIGURE



## References

[1] Takahashi K, Yamanaka S (2006). Induction of pluripotent stem cells from mouse embryonic and adult fibroblast cultures by defined factors. Cell.

[2] Compagnucci C, Nizzardo M, Corti S, Zanni G, Bertini E (2014). In vitro neurogenesis: development and functional implications of iPSC technology. Cell Mol Life Sci.

[3] Bukowiecki R, Adjaye J, Prigione A (2014). Mitochondrial function in pluripotent stem cells and cellular reprogramming. Gerontology.

[4] Raikwar SP, Kim EM, Sivitz WI, Allamargot C, Thedens DR, Zavazava N (2015). Human iPS cell-derived insulin producing cells form vascularized organoids under the kidney capsules of diabetic mice. PLoS One.

[5] Beckmann A, Schubert M, Hainz N, Haase A, Martin U, Tschernig T, Meier C (2016). Ultrastructural demonstration of Cx43 gap junctions in induced pluripotent stem cells from human cord blood. Histochem Cell Biol.

[6] Liang G, Zhang Y (2013). Genetic and epigenetic variations in iPSCs: potential causes and implications for application. Cell Stem Cell.

[7] Masotti A, Celluzzi A, Petrini S, Bertini E, Zanni G, Compagnucci C (2014). Aged iPSCs display an uncommon mitochondrial appearance and fail to undergo in vitro neurogenesis. Aging (Albany NY).

[8] Petrini S, Borghi R, D'Oria V, Restaldi F, Moreno S, Novelli A, Bertini E, Compagnucci C (2017). Aged induced pluripotent stem cell (iPSCs) as a new cellular model for studying premature aging. Aging (Albany NY).

[9] Narayan K, Subramaniam S (2015). Focused ion beams in biology. Nat Methods.

[10] Kizilyaprak C, Daraspe J, Humbel BM (2014). Focused ion beam scanning electron microscopy in biology. J Microsc.

[11] Raposo G, Stoorvogel W (2013). Extracellular vesicles: exosomes, microvesicles, and friends. J Cell Biol.

[12] Pankiv S, Clausen TH, Lamark T, Brech A, Bruun JA, Outzen H, Øvervatn A, Bjørkøy G, Johansen T (2007). p62/SQSTM1 binds directly to Atg8/LC3 to facilitate degradation of ubiquitinated protein aggregates by autophagy. J Biol Chem.

[13] Takahashi K, Yamanaka S (2016). A decade of transcription factor-mediated reprogramming to pluripotency. Nat Rev Mol Cell Biol.

[14] Chhabra A (2017). Derivation of Human Induced Pluripotent Stem Cell (iPSC) Lines and Mechanism of Pluripotency: Historical Perspective and Recent Advances. Stem Cell Rev.

[15] Martin U (2017). Genome stability of programmed stem cell products. Adv Drug Deliv Rev.

[16] Suhr ST, Chang EA, Tjong J, Alcasid N, Perkins GA, Goissis MD, Ellisman MH, Perez GI, Cibelli JB (2010). Mitochondrial rejuvenation after induced pluripotency. PLoS One.

[17] Di Giulio A, Maurizi E, Stacconi MV, Romani R (2012). Functional structure of antennal sensilla in the myrmecophilous beetle Paussus favieri (Coleoptera, Carabidae, Paussini). Micron.

[18] Di Giulio A, Muzzi M, Romani R (2015). Functional anatomy of the explosive defensive system of bombardier beetles (Coleoptera, Carabidae, Brachininae). Arthropod Struct Dev.

[19] Wang Y, Zhang L, Li Y, Chen L, Wang X, Guo W, Zhang X, Qin G, He SH, Zimmerman A, Liu Y, Kim IM, Weintraub NL, Tang Y (2015). Exosomes/microvesicles from induced pluripotent stem cells deliver cardioprotective miRNAs and prevent cardiomyocyte apoptosis in the ischemic myocardium. Int J Cardiol.

[20] Jung JH, Fu X, Yang PC (2017). Exosomes Generated From iPSC-Derivatives: New Direction for Stem Cell Therapy in Human Heart Diseases. Circ Res.

[21] Wu J, Qu Z, Fei ZW, Wu JH, Jiang CP (2017). Role of stem cell-derived exosomes in cancer. Oncol Lett.

[22] Lonergan T, Bavister B, Brenner C (2007). Mitochondria in stem cells. Mitochondrion.

[23] Passos JF, Nelson G, Wang C, Richter T, Simillion C, Proctor CJ, Miwa S, Olijslagers S, Hallinan J, Wipat A, Saretzki G, Rudolph KL, Kirkwood TB, von Zglinicki T (2010). Feedback between p21 and reactive oxygen production is necessary for cell senescence. Mol Syst Biol.

[24] Panopoulos AD, Yanes O, Ruiz S, Kida YS, Diep D, Tau-tenhahn R, Herrerías A, Batchelder EM, Plongthongkum N, Lutz M, Berggren WT, Zhang K, Evans RM (2012). The metabolome of induced pluripotent stem cells reveals metabolic changes occurring in somatic cell reprogramming. Cell Res.

[25] Zhang J, Nuebel E, Daley GQ, Koehler CM, Teitell MA (2012). Metabolic regulation in pluripotent stem cells during reprogramming and self-renewal. Cell Stem Cell.

[26] Youle RJ, Narendra DP (2011). Mechanisms of mitophagy. Nat Rev Mol Cell Biol.

[27] Fîlfan M, Sandu RE, Zăvăleanu AD, GreşiŢă A, Glăvan DG, Olaru DG, Popa-Wagner A (2017). Autophagy in aging and disease. Rom J Morphol Embryol.

[28] Gaspar-Maia A, Alajem A, Meshorer E, Ramalho-Santos M (2011). Open chromatin in pluripotency and reprogramming. Nat Rev Mol Cell Biol.

[29] Vazquez-Martin A, Van den Haute C, Cufí S, Corominas-Faja B, Cuyàs E, Lopez-Bonet E, Rodriguez-Gallego E, Fernández-Arroyo S, Joven J, Baekelandt V, Menendez JA (2016). Mitophagy-driven mitochondrial rejuvenation regulates stem cell fate. Aging (Albany NY).

[30] Hung SS, Van Bergen NJ, Jackson S, Liang H, Mackey DA, Hernández D, Lim SY, Hewitt AW, Trounce I, Pébay A, Wong RC (2016). Study of mitochondrial respiratory defects on reprogramming to human induced pluripotent stem cells. Aging (Albany NY).

[31] Wong RC, Lim SY, Hung SS, Jackson S, Khan S, Van Bergen NJ, De Smit E, Liang HH, Kearns LS, Clarke L, Mackey DA, Hewitt AW, Trounce IA, Pébay A (2017). Mitochondrial replacement in an iPSC model of Leber's hereditary optic neuropathy. Aging (Albany NY).

[32] Crombie DE, Curl CL, Raaijmakers AJ, Sivakumaran P, Kulkarni T, Wong RC, Minami I, Evans-Galea MV, Lim SY, Delbridge L, Corben LA, Dottori M, Nakatsuji N (2017). Friedreich's ataxia induced pluripotent stem cell-derived cardiomyocytes display electrophysiological abnormalities and calcium handling deficiency. Aging (Albany NY).

[33] Gough NR, Fambrough DM (1997). Different steady state subcellular distributions of the three splice variants of lysosome-associated membrane protein LAMP-2 are determined largely by the COOH-terminal amino acid residue. J Cell Biol.

[34] Janvier K, Bonifacino JS (2005). Role of the endocytic machinery in the sorting of lysosome-associated membrane proteins. Mol Biol Cell.

[35] Krishnan V, Bane SM, Kawle PD, Naresh KN, Kalraiya RD (2005). Altered melanoma cell surface glycosylation mediates organ specific adhesion and metastasis via lectin receptors on the lung vascular endothelium. Clin Exp Metastasis.

[36] Tamada H, Kiryu-Seo S, Hosokawa H, Ohta K, Ishihara N, Nomura M, Mihara K, Nakamura KI, Kiyama H (2017). Three-dimensional analysis of somatic mitochondrial dynamics in fission-deficient injured motor neurons using FIB/SEM. J Comp Neurol.

[37] Do T, Murphy G, Earl LA, Del Prete GQ, Grandinetti G, Li GH, Estes JD, Rao P, Trubey CM, Thomas J, Spector J, Bliss D, Nath A (2014). Three-dimensional imaging of HIV-1 virological synapses reveals membrane architectures involved in virus transmission. J Virol.

