# Supplementary material for: Senescence-associated ultrastructural features of long-term cultures of induced pluripotent stem cells (iPSCs)
Source: Aging (Albany NY). 2017 Oct 23;9(10):2209–22. doi: 10.18632/aging.101309 (PMC5680563; doi:10.18632/aging.101309)
Supplement: Supplementary file 1 [file aging-09-2209-s001.pdf]

SUPPLEMENTARY MATERIAL

Supplementary experimental procedures

Analyses of the fluorescence intensity

The mean pixel values (as an indication of the fluorescence intensity) were measured from at least five images (including from ~70 to ~200 cells) for each antibody. Images (obtained using TCS-SP8X laser-scanning confocal microscope, Leica Microsystems, Mannheim, Germany) were uploaded to ImageJ and the intensity of fluorescence for each image was converted to a single numeric value using the RGB color model. Individual red, green and blue (RGB) values were generated for each image using ImageJ and then converted to a singular gray scale value. The resulting gray scale value represents overall intensity (Solomon C, Breckon T. Fundamentals of digital image pro-cessing: a practical approach with example in Matlab. Chichester, U.K.: Wiley-Blackwell, 2011). The mean pixel value was obtained by analyzing five images for each sample, having from 70 to 200 cells per image.

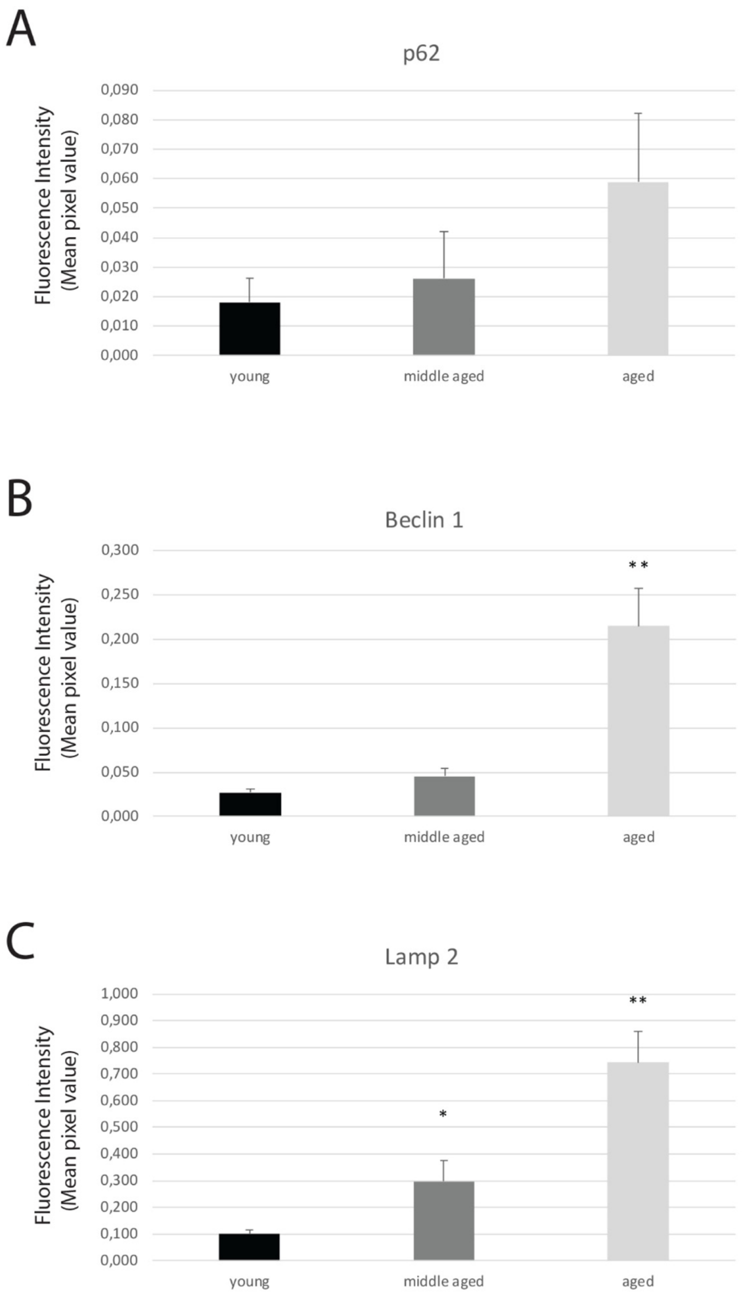

Supplementary Figure 1. Mean pixel value for p62 (**A**), Beclin 1 (**B**) and Lamp 2 (**C**). The y axis represents the grayscale value of the signal measured from the immunofluorescence image (obtained with Leica confocal microscope).
